# Impact of cryoprotectant-free sperm vitrification in pulled-glass capillary on sperm parameters and DNA integrity: A lab trial study

**DOI:** 10.18502/ijrm.v22i4.16391

**Published:** 2024-06-12

**Authors:** Minh Tam Le, Trung Van Nguyen, Thai Thanh Thi Nguyen, Hong Nhan Thi Dang, Quoc Huy Vu Nguyen

**Affiliations:** ^1^Center for Reproductive Endocrinology and Infertility, Hue University of Medicine and Pharmacy, Hue University, Hue City, Vietnam.; ^2^Department of Obstetrics and Gynecology, Hue University of Medicine and Pharmacy, Hue University, Hue City, Vietnam.

**Keywords:** Vitrification, Sperm, DNA fragmentation, Cryoprotectant agents, Small volume.

## Abstract

**Background:**

Vitrification is a recently introduced yet widely applied assisted reproduction technique. So far, the effects of the chemicals and devices in vitrification on sperm motility and DNA integrity are still unclear.

**Objective:**

This study aimed to examine sperm quality, as determined by semen analysis and sperm DNA integrity when vitrified with or without cryoprotectant agents (CPAs) using pulled-glass capillaries.

**Materials and Methods:**

Between February and June 2020, 50 infertile men from the Hue Center for Reproductive Endocrinology and Infertility, Hue University of Medicine and Pharmacy, Vietnam, were enrolled. Sperm samples, prepared using the swim-up technique, were divided into 2 groups: vitrification with CPAs (group 1) and without CPAs (group 2). Vitrified sperm samples were preserved in 10 µL pulled-glass capillaries. Motility, sperm membrane integrity, and the DNA fragmentation index were tested.

**Results:**

Sperm motility in vitrified media with CPAs (54.4 ± 11%) was statistically higher than in media without CPAs (51.14 ± 10.6%, p < 0.05). CPAs did not affect sperm membrane integrity or large halo ratio (71.34 ± 8.47 vs. 70.38 ± 8.11 and 50.84 ± 18.92 vs. 51.98 ± 19.44, respectively). Group 2 exhibited a lower DNA fragmentation index than group 1 after vitrification (14.2 ± 8.47 vs. 12.60 ± 9.03, p = 0.021).

**Conclusion:**

Using a pulled-glass capillary for sperm vitrification, the presence of CPAs in the vitrification medium resulted in higher progressive motility and lower DNA fragmentation index than the medium without CPAs.

## 1. Introduction

Cryopreservation of human spermatozoa is a popular technique used in assisted reproduction. It preserves the male reproductive gamete, making it useful for donor sperm banks, oncologic therapies, and male infertility treatment (1). For sperm cryoprotection, techniques ranging from slow to rapid and ultrafast cooling are available (2).

In conventional freezing, semen is mixed with permeable or impermeable cryoprotectant agents (CPAs), preventing intracellular ice formation (3, 4). However, permeable CPAs can induce osmotic changes, leading to extensive cell shrinkage and mechanical damage (5, 6). Additional cell penetration of permeable CPAs can alter lipid-phase transitions and increase lipid peroxidation. This phenomenon results in extensive chemical and physical damage to the cell membranes of spermatozoa, as well as structural damage to the cytoskeleton and antioxidant enzymes (7).

Sperm vitrification can be combined with permeable or impermeable CPAs in reduced storage volumes (8). Studies have been conducted on the vitrification of animal and human spermatozoa for volumes ranging from 30–500 µL (3, 7). Although using permeable CPAs such as glycerol has been approved as a viable option for sperm vitrification (9), this supplementation can negatively affect sperm. The use of impermeable CPAs containing proteins (human serum albumin) and carbohydrates (sucrose, raffinose, and trehalose) for dehydration and stabilization of the cellular membrane are recommended for cryopreservation techniques that cause less damage to important sperm functions (2). Thus, permeable CPA-free vitrification has great potential for the cryopreservation of human sperm for assisted reproduction (7, 9).

However, some studies have reported that impermeable CPAs, such as sucrose, have detrimental effects on the DNA quality of sperm (7, 10, 11). Specifically, chromatin and DNA damage are more significant in the vitrified sperm group with sucrose than in the group without sucrose. Although impermeable CPAs can prevent chemical toxicity during vitrification and warming, osmotic shock can result in undesirable side effects. Sperms in micro volumes were vitrified theoretically based on their physiological features. Spermatozoa are tiny and contain significant amounts of proteins and carbohydrates, which may act as natural CPAs to prevent intracellular ice crystal formation during cooling and reheating. In addition, when a microvolume of the mixture is applied, spermatozoa rapidly transform from liquid to stable glass form, preventing the formation of intracellular and extracellular ice crystals. During vitrification without CPAs, sperm are not subjected to the high osmotic pressure of the cryopreservation medium, which can result in excessive sperm cell shrinkage due to the movement of water and CPAs into and out of the cell during vitrification and warming (10).

The vitrification of spermatozoa in volumes as tiny as 30 µL has been studied to enhance the effectiveness of sperm storage. Studies that preserve sperms utilizing a funnel grid, cell freezing, or cryotop vitrification equipment are essential (2, 11). These procedures can effectively increase sperm vitrification. However, certain negative qualities still need improvement (12, 13).

This study aimed to determine the variations in sperm cryopreservation by measuring sperm motility and DNA fragmentation using a medium with or without CPAs and tiny volume glass capillaries.

## 2. Materials and Methods

**Figure 1:**
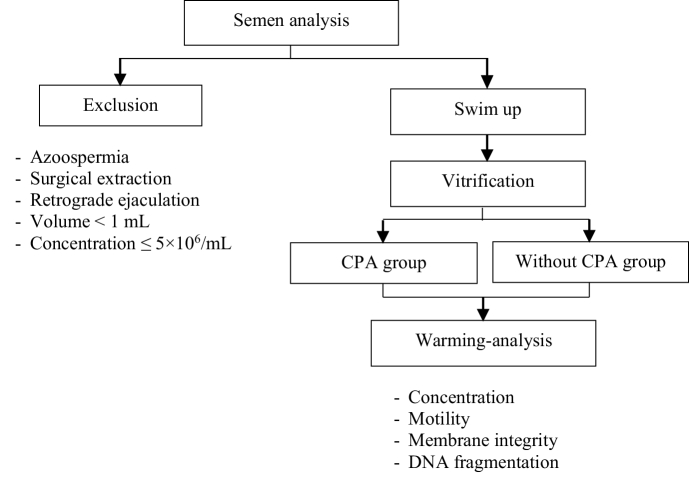
Flowchart diagram of study design. CPA: Cryoprotectant agent.

This lab trial study focused on sperm from 50 infertile men referred for fertility evaluation at the Hue Center for Reproductive Endocrinology and Infertility, Hue University of Medicine and Pharmacy, Vietnam, between February and June 2020. After employing the swim-up technique for sample preparation, each sperm sample was subsequently divided into 2 halves: the first group (group 1, n = 50) underwent vitrification in media containing CPAs, while the second group (group 2, n = 50) underwent vitrification in media without CPA. The research diagram is shown in figure 1.

Following the 2010 World Health Organization recommendations for the analysis of human sperm, semen samples were tested and categorized. Inclusion criteria included men who had sperm concentrations more than 5 x 10^6^/mL. Inability to ejaculate, sperm obtained through surgical extraction, retrograde ejaculation, semen volume < 1 mL, severe oligozoospermia, or azoospermia were excluded from the study.

A Primo Star microscope (Zeiss, Jena, Germany) was used to observe and classify sperm motility as either progressive or non-progressive. Sperm with increasing motility moved quickly in a straight line or a big circle. Despite displaying apparent flagella movement, sperm with non-progressive motility moved in a small circle.

The sperm concentration was measured using a 20-fold dilution of semen sample with fixative and sperm count on an improved Neubauer counting chamber.

Using the eosin Y technique, the sperm vitality test determined whether sperm were alive or dead. Spermatozoa with intact membranes could prevent dye penetration and were categorized as alive. Spermatozoa that exhibited pink/red staining were classified as nonviable or membrane impaired. At × 400 magnification, 200 spermatozoa were evaluated on each slide (14). The sperm morphology test utilized the Giemsa stain procedure and was observed by the Primo Star microscope (Zeiss) at × 1000 magnification to analyze the morphology of the head, acrosome region, sperm neck, mid-piece, tail, and cytoplasmic droplets. At least 200 spermatozoa were counted to determine the proportion of spermatozoa with normal and abnormal morphology. When the rate of normal spermatozoa falls below 4%, abnormal morphology is recorded.

The Halosperm test, based on the sperm chromatin dispersion technique, was carried out using the HalospermⓇ kit (Halotech, Madrid, Spain) following the manufacturer's instructions. Human sperm DNA fragmentation was classified as follows: a) Big/big halo: halo width ≥ core diameter, b) Medium halo: small halo ≤ medium halo ≤ large halo, c) Small halo: halo width ≤ 1/3 of the core diameter, d) Without halo: absence of halo, and e) Degraded: absence of halo and unevenly or weakly stained core.

500 sperms were counted during the DNA fragmentation assessment (15). This formula calculated the DNA fragmentation index (DFI): 


$$DFI=\frac{Smallhalo+Nohalo+Degenerate}{Totalspermcount}\times 100$$

The DFI threshold is at 30% to distinguish between 2 groups: abnormal DFI ( ≥ 30%) and normal DFI ( < 30%).

The results were calculated as the average obtained by 2 technicians. ImageJ software (a Java-based image processing program developed at the National Institutes of Health and the Laboratory for Optical and Computational Instrumentation) was used to capture photographs and measure the sizes of the halos in the sperm DNA fragmentation assay.

### Semen preparation

The swim-up method was utilized to prepare semen. First, 1 mL of sperm was placed in a 14 mL round-bottom tube, and a 1 mL layer of Flushing medium (FertiProⓇ, Beermem, Belgium) that had been previously heated at 37 C was carefully deposited on top. After this, the tube was placed at an angle of 45 degrees in an incubator at 37 C for 30 min.

To prevent the mixing of seminal plasma, the supernatant was collected gently and centrifuged at 350 g for 7 min in a 15 mL tube with a pointed bottom. After the supernatant was discarded, the silt was dissolved in 100 µL of 37 C-preheated flushing medium. By adding 5 µL of sperm to the improved Neubauer counting chamber, the concentration and motility of swim-up-prepared sperm were examined.

### Sperm vitrification

After preparation, semen samples were divided into 2 halves. The first half (without the CPA group) was vitrified immediately, and the second (CPA group) was diluted with SpermFreeze solution (VitrolifeⓇ, V. Frolunda, Sweden) in a ratio of 1:1. The sperm sample was retained for 5 min to equalize the osmotic pressure and then vitrified.

SpermFreeze solution contains 12% glycerol (CPA) for freezing and has continued to be frequently utilized in vitrification processes using various materials, including cryoloops, cryotop, and cryoleaf (13). A storage volume of 10 µL was employed for both samples with and without CPA; for samples without CPA, the 10 µL swim-up was loaded, but for samples with CPA, 5 µL of the sample was combined with 5 µL of CPA. In in-vitro fertilization, pulled glass capillary is widely used to remove cumulus cells from oocytes. In order to use a stereomicroscope at x25 magnification, the homogeneity of the capillary diameter was compared to that of the standard denude pipette (Vitrolife).

**Figure 2:**
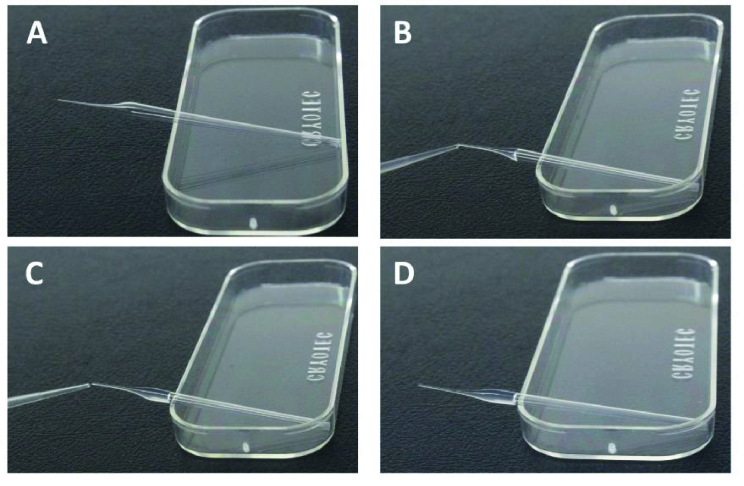
Loading sperm to pulled capillary for vitrification. A) Pulled capillary. B) Using a micropipette and tip to load 10 µl sample into the pulled capillary's lumen. C) All the sample was easily loaded into the lumen by capillary force. D) Pulled capillary after loading the sample.

For the group without CPA, the pulled-glass capillary technique was conducted using a Pasteur pipette (KimbleⓇ    , Milville, USA) with an inner diameter of 135 µm and a length of 3 cm, which was adequate for manipulating a sample volume of 10 µL (Figure 2). The pulled-glass capillary containing the sample was put on an open surface 1 cm above liquid nitrogen to cool the sample rapidly. After 2 min, the microcapillary was immediately stored in liquid nitrogen (13, 14).

For the CPA group: A similar pulled-glass capillary was utilized to withdraw 10 µL of sperm mixture diluted in SpermFreeze solution and then placed on an open surface 1 cm above liquid nitrogen for 2 min to cool the sample rapidly. The cooled capillary was then immediately immersed in liquid nitrogen for vitrification.

The immersed pulled-glass capillaries were afterward collected, placed in a Nunc cryovial tube (Nunc CryoTubes^TM^, Guangdong, China), connected to a cryocane (MTG, Munster, Germany), and stored in liquid nitrogen tanks (13). Cryovial tubes are routinely used to store vitrified human spermatozoa.

### Warming stage

After 7 days, the pulled-glass capillary was taken from the liquid nitrogen storage tank and placed in a container containing liquid nitrogen. The capillary was dipped in a 1.5 mL tube of Flushing media, which was heated at 37 C for 30 sec to melt the sample (13, 16). After completely submerging the capillary in the liquid, it was gently agitated. The sample was placed in a droplet holding 10 µL of flushing media, covered with oil, and incubated at 37 C after liquefaction.

After warming the sperm, the motility, membrane integrity, and DNA fragmentation were reevaluated. This material was combined, and 5 µL was extracted for concentration and mobility analysis using an improved Neubauer counting chamber. The membrane integrity was next evaluated by loading 1 µL of the sample into 2 µL drops of Eosin dye (Merck KgaA, Gernsheim, Germany) (17). The DNA fragmentation of a 2 µL sample diluted with 6 µL agarose solution was reevaluated.

The sperm quality assessment before and after vitrification was performed by 2 well-trained embryologists with over 10 yr of expertise, and the acceptable deviation between their results was less than 10%.

### Ethical considerations

This study was approved by the Ethics Committee of Hue University of Medicine and Pharmacy, Hue, Vietnam (Code: H2019/010). All methods were performed in accordance with the relevant guidelines of assisted reproductive techniques. Informed consent was obtained from all participants following the Declaration of Helsinki.

### Statistical analysis

The IBM SPSS version 20.0 (IBM Corp., Armonk, NY, USA) was utilized for the analysis of research data. Variables were described using mean ± SD and percentages (%). Pairwise comparisons were employed to assess the quality of semen samples before and after preparation, as well as the quality of samples after vitrification under different conditions. Statistical significance was determined with p < 0.05.

## 3. Results

The general characteristics of the study population are shown in table I. The mean age was 34.84 ± 6.4 yr. The semen was collected after 4.28 ± 1.18 days of sexual abstinence. The mean progressive motility of sperm, the mean concentration, the mean vitality, and the mean normal morphology were observed before preparation. The DFI was relatively high (25.37 ± 17.76%) and broadly distributed.

The swim-up method of sperm preparation allowed us to choose better sperm with higher motility and DNA integrity. Table II indicates these changes in sperm progressive motility and large halo sperm were statistically significant after sperm preparation with a p < 0.001.

Table III presents the results of vitrification and warming on sperm motility. The rate of progressive sperm motility in the CPA group was significantly higher than the group without CPA (p = 0.027). Specifically, in the subgroup of fresh samples with abnormal motility (before vitrification), the rate of progressive motility after vitrification in the CPA group sperm was greater than that of without CPA group sperms.

After warming, the plasma membrane integrity rate, in both the groups (with and without CPA), was found to be high. The statistical difference between the 2 groups was not significant. The DFI in the CPA group was significantly higher than in the without CPA group (p = 0.021).

**Figure 3:**
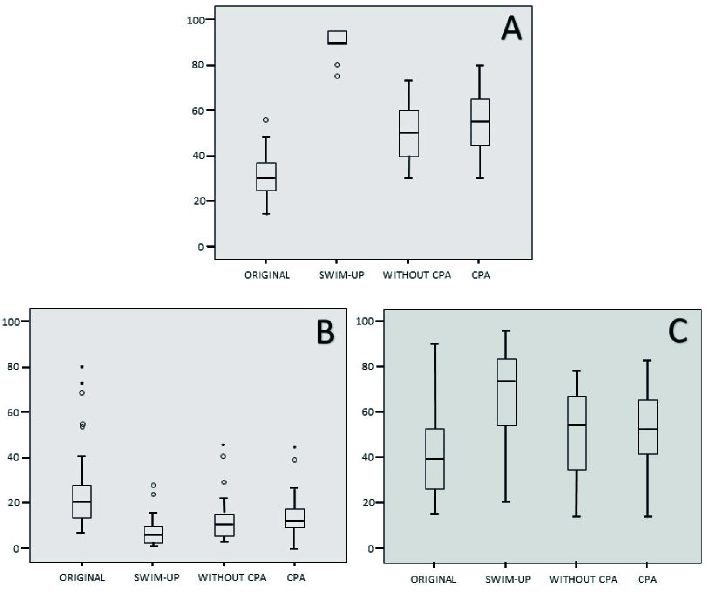
Sperm motility and DNA fragmentation before and after vitrification in a medium with or without cryoprotective agents. A) Distribution of progressive motility of spermatozoa. B) Distribution of sperm DNA fragmentation index. C) Distribution of large halo ratio.

After vitrification and warming, no significant difference between the CPA and without CPA group regarding the large halo ratio was observed. Figure 3 presents sperm motility and DNA fragmentation before and after vitrification in a medium with or without cryoprotective agents.

**Table 1:** General characteristics of the study population


**Variables**	**Mean ± SD**	**Median**	**Min**	**Max**	**Interquartile range**
**Age (yr)**	34.84 ± 6.4	34.00	25	53	9.50
**Sexual abstinence (days)**	4.28 ± 1.18	4.00	3	7	2.00
**Volume (mL)**	2.46 ± 0.94	2.00	1.0	5.5	1.00
**pH**	7.23 ± 0.4	7.00	6.5	8.5	0.50
**PR motility (%)**	31.1 ± 8.59	30.00	14	56	12.25
**Non-PR motility (%)**	29.18 ± 6.69	29.00	18	44	9.25
**Immobility (%)**	39.72 ± 9.15	38.00	24	62	8.50
**Concentration (mil/mL)**	38 ± 17.14	37.50	15	89	25.25
**Vitality (%)**	84.16 ± 5.96	84.50	68	93	9.00
**Normal morphology**	3.54 ± 1.54	3.00	1	8	2.25
**DNA damage (DFI%)**	25.37 ± 17.76	20.50	6.4	81	13.95
**< 30% (n = 41)**	18.24 ± 6.53	17.00	6.4	29	12.20
**≥ 30% (n = 9)**	57.82 ± 16.54	54.80	37.2	81	32.80
DFI: DNA fragmentation index, PR: Progressive					

**Table 2:** The results of sperm parameters after preparation by swim-up


**Variables **	**Original**	**Swim-up**	**P-value**
**Progressive motility (%)**	31.1 ± 17.14 (30.00, 12.25)	90.30 ± 6.18 (90.00, 5.00)	< 0.001
**Concentration (mil/mL)**	38 ± 17.14 (7.50, 15.25)	26.22 ± 11.88 (25.00, 15.25)	< 0.001
**Total progressive motility (mil)**	3125.9 ± 2684.9 (2468.75, 2474.25)	239.1 ± 114.8 (237.50, 147.00)	< 0.001
**DNA damage (DFI%)**	25.37 ± 17.76 (20.50, 13.95)	7.84 ± 6.7 (6.00, 7.00)	< 0.001
**DFI < 30%**	18.24 ± 6.53 (17.00, 12.20)	6.71 ± 6.01 (5.00, 4.50)	< 0.001
**DFI ≥ 30%**	57.82 ± 16.54 (54.80, 32.80)	13 ± 7.59 (13.00, 12.00)	< 0.001
**Large halo (%)**	43.64 ± 19.78 (39.40, 26.85)	71.1 ± 17.37 (74.00, 29.25)	< 0.001
Data are presented as Mean SD (median, interquartile range). Paired-sample *t* test. DFI: DNA fragmentation index			

**Table 3:** Effect of vitrification with or without cryoprotective agents on sperm motility and DNA fragmentation


**Variables**	**CPAs**	**Without CPA**	**P-value**
**Concentration (mil/mL)**	16.14 ± 5.31 (15.00, 6.50)	20.76 ± 6.25 (20.00, 7.75)	< 0.001
**Progressive motility (%)**	54.4 ± 11.09 (55.00, 20.00)	51.14 ± 10.6 (50.00, 20.00)	0.027
**PR < 32%**	51.72 ± 9.75 (50.00, 12.50)	48.51 ± 9.97 (50.00, 15.00)	0.045
**PR ≥ 32%**	58.09 ± 11.99 (60.00, 15.00)	54.76 ± 10.61 (55.00, 20.00)	0.233
**Total progressive motility (mil)**	19.5 ± 7.7 (17.58, 9.94)	23.8 ± 9.3 (21.50, 13.71)	< 0.001
**Membrane integrity (%)**	71.34 ± 8.47 (71.00, 10.25)	70.38 ± 8.11 (69.00, 9.50)	0.294
**DFI (%)**	14.2 ± 8.47 (12.00, 8.25)	12.60 ± 9.03 (10.50, 9.00)	0.021
**DFI < 30%**	12.95 ± 6.67 (12.00, 11.00)	11.63 ± 6.74 (11.00, 9.00)	0.089
**DFI ≥ 30%**	19.89 ± 13.14 (17.00, 19.00)	17 ± 15.67 (9.00, 23.50)	0.081
**Large halo (%)**	50.84 ± 18.92 (52.00, 26.00)	51.98 ± 19.44 (54.50, 33.00)	0.350
Data are presented as Mean ± SD (median, interquartile range). Paired-sample *t* test. CPAs: Cryoprotectant agents, DFI: DNA fragmentation index, PR: Progressive			

## 4. Discussion

Vitrification of spermatozoa is the preferred method for sperm preservation, and small-volume storage can be used to rapidly freeze and thaw sperms with 100% recovery despite the container's impact on the storage contents (12). In this study, small amounts of sperm samples were kept in a pulled-glass capillary to investigate the impact of CPAs on vitrification media on sperm quality.

In vitrification, a small sample can be rapidly cooled to prevent the development of ice crystals (11, 12). The smallest containers for sperm vitrification can be manufactured from biological and non-biological substances. However, applying aseptic methods and handling small volumes is challenging, particularly when using the funnel grid system. Cryotop is currently utilized for the vitrification of embryos, although it is costly, and sperm may adhere to the cryotop's wall after warming. In addition, the container must be periodically washed because of greasy medium droplets (12, 13). To load sperms into this manipulator system, additional devices are necessary due to the complexity of the loading procedure. In general, financial concerns have impeded their widespread application.

Sperm may be vitrified in capillary glass vessels with a progressive motility of 67.8% after warming (8). In our investigation, spermatozoa were stored in a pulled-glass capillary with a diameter of approximately 135 µm following the vitrification methodology. The sperm samples were maintained in capillary tubes with a small diameter and were always contained within the lumen, minimizing sperm loss during the procedure. During vitrification and warming, the section of the pulled capillary increases the sample's heat exchange potential. Because these capillary tubes are composed of glass, their thickness and toughness allow them to withstand manipulation and heat transfer without fracturing. In addition, sperm are easily manipulable during warming, making it simple to add them to a little drop of intra-cytoplasmic sperm injection media and examine them under a microscope during the operation (3, 12).

The motility of spermatozoa following vitrification is a crucial characteristic. Progressive sperm motility guarantees that sperms possess virtually no cell damage, intact microtubule structure, and intact mitochondria (13). To achieve a high rate of progressive motility, we utilized swim-up-prepared sperm samples. Similar to earlier research, the percentage of motile spermatozoa reduced dramatically after vitrification and warming (2, 3, 7).

The sperm motility in vitrified media with CPA was greater than that in vitrified media without CPA in the motility group (below 32%), and not for those with normal motility. In the aberrant motility group, the proportion of motile sperm in the vitrification group with CPA was substantially greater than in the group without CPA. This result is similar to previous studies that reported that sperm samples vitrified with CPA had a significantly higher proportion of sperm motility and viability than those vitrified without CPA (11, 17). Adding CPAs to sperms with faulty motility mitigates the effect of cold damage on motility in sperms with abnormal motility.

In our study, the plasma membrane integrity rate was 71.34% among CPAs and 70.38% among those without CPAs, with no statistically significant difference between the 2 groups. Thus, within a modest volume of cryopreservation, sperm membrane damage can be avoided without adding CPA to the vitrification media. According to previous research (2, 3, 18), the integrity of sperm plasma membranes ranged from 56–70% after warming, after vitrification. CPAs influence the permeability of the sperm membrane, hence decreasing the intracellular osmotic volume. There have been reports of cell shrinkage and irreversible damage to the integrity of the sperm plasma membrane and other ultrastructural organelles (17). Osmotic shock is commonly recognized as the most detrimental effect of cryopreservation. When 0.5 M sucrose (impermeable CPA) is concentrated with culture media, the osmotic pressure is 536 mOsm/kg (19). In addition to intracellular and extracellular CPAs, plasma macromolecules were used to improve the efficiency of sperm vitrification. As Nabavinia's study showed a positive effect on sperm motility, viability, and DNA integrity by adding a platelet-rich plasma product, they concluded that platelet-rich plasma has a beneficial impact on sperm (20).

Ensuring sperm DNA integrity is crucial to sperm cryopreservation processes (13). The halosperm test is an effective method for evaluating the packing and preservation of sperm DNA after cryopreservation. During cryopreservation, the vitrified and warming stages can lead to protein breakdown and increased protein composition (16). Protein degradation, post-translational processing, secondary or tertiary structure variations, or transfer to other cellular compartments contribute to spermatozoa protein level changes. Our research centered on 2 crucial sperm DNA quality parameters: DFI and big halo ratio. Large halo rings could only be produced by vitrified and warmed spermatozoa with DNA integrity. Recent research from the United Kingdom using the halosperm test method to measure sperm DNA fragmentation demonstrated that vitrification is superior to conventional freezing (19). DFI indicates the proportion of DNA fragments, and a large halo represents the ratio of sperm with the most intact DNAs.

In our study, the DFI in the CPA group (14.2 ± 8.47%) was significantly higher than that in the group without CPA (12.60 ± 9.03%) with a p = 0.021. However, the large halo ratio results did not reveal any significant difference between the CPA and non-CPA groups (50.84% vs. 51, 98%). This suggests that employing a CPA medium in a glass capillary with a tiny volume does not preserve sperm DNA from harm. This finding is similar to a recent research that used a 0.5 µL sample placed on a Cryotop (Kitazato, Tokyo, Japan) polypropylene strip for sperm vitrification (10).

### Limitations

The quality of sperm was assessed in our study through the utilization of Giemsa and eosin stain. The effectiveness of the Papanicolaou stain in assessing sperm morphology is well acknowledged, mostly attributed to its ability to impart distinct colors to individual sperm components. While the Giemsa technique remains unmentioned in the World Health Organization's guidelines for assessing the morphological quality of human sperm, some published studies continue to use it. The aforementioned approach successfully distinguished discrete morphological characteristics of sperm, encompassing its head, acrosome, vacuole, neck, and tail. The results achieved using Giemsa continue to be positive and are considered a quick, uncomplicated, and economical method. Although the Eosin-Nigrosin method is commonly employed to evaluate sperm viability and membrane integrity, the 2010 World Health Organization recommends that eosin alone can also be used to ensure an accurate assessment of sperm vitality. On page 29, the instructions specify the sperm sample to be examined only by the Eosin procedure, with a magnification ranging from 200–400X. These practices, however, should be regarded as limitations of our research.

## 5. Conclusion

In conclusion, the pulled-glass capillaries can be used to vitrify human spermatozoa due to their efficiency and convenience. Using this device, the presence of CPAs in the vitrification solution resulted in higher progressive motility and lower DFI than the absence of CPAs. After vitrification and warming, no difference was observed in plasma membrane integrity and DNA integrity between groups with and without CPAs.

##  Data availability

The data supporting the results of this study are available upon request from the corresponding author, Minh Tam Le by email: leminhtam@huemed-univ.edu.vn or leminhtam@hueuni.edu.vn.

##  Author contributions

Minh Tam Le, Trung Van Nguyen, Thai Thanh Thi Nguyen, Hong Nhan Thi Dang had full access to all of the data in the study and takes responsibility for the integrity of the data and the accuracy of the data analysis. Concept and design Minh Tam Le, Trung Van Nguyen, Thai Thanh Thi Nguyen. Acquisition, analysis, or interpretation of data Minh Tam Le, Trung Van Nguyen, Thai Thanh Thi Nguyen, Hong Nhan Thi Dang. Drafting of the manuscript: All authors. Critical revision of the manuscript for important intellectual content. All authors. Statistical analysis: Trung Van Nguyen, Thai Thanh Thi Nguyen, Hong Nhan Thi Dang. Supervision: Minh Tam Le, Quoc Huy Vu Nguyen.

##  Conflict of Interest

The authors declare that there is no conflict of interest.
